# An Analysis of Acceleration, Deceleration and High-Intensity Skating during Elite Bandy Match-Play: A Case Study

**DOI:** 10.3390/sports9110152

**Published:** 2021-11-10

**Authors:** Malin Johansson, Per-Erik Ervasti, Sven Blomqvist

**Affiliations:** Department of Public Health and Sports Science, Faculty of Health and Occupational Studies, University of Gävle, Kungsbäcksvägen 47, SE-801 76 Gävle, Sweden; per-erik.ervasti@hig.se (P.-E.E.); sven.blomqvist@hig.se (S.B.)

**Keywords:** physical workload, time-dependent reduction, accumulated fatigue

## Abstract

Profiles of physical workload in sports are useful to optimize performance and reduce the risk of injury. The aim of the study was to investigate physical workload in 10 elite bandy players by describing acceleration, deceleration, and high-intensity skating during bandy match-play. During 13 home matches, 10 male elite bandy players wore a GPS unit to measure changes in the total distance, total distance skating in two speed zones, and total distance of acceleration and deceleration. A within-subject design was used to measure changes over time during match-play by comparing first and second halves as well as comparisons for consecutive 15 min intervals. No significant differences were observed for high-intensity or very high-intensity acceleration and deceleration for comparisons by halves or for 15-min intervals. For comparisons by halves, a significant time-dependent effect was observed on very fast skating (1337.6 m vs. 1160.9 m), sprint skating (300.0 m vs. 272.0 m), low-intensity acceleration (342.7 m vs. 333.0 m), and total distance covered (10,916.9 m vs. 10,450.3 m). These variables, along with low-intensity deceleration, were also significant for the 15 min interval comparisons. The results show that there is no time-dependent reduction in high-intensity acceleration and deceleration in elite bandy match-play. However, elite bandy players do not maintain the distance of high-intensity skating throughout a whole match.

## 1. Introduction

Profiles of the physical workload in different sports are useful for coaches to optimize performance on both the team and individual levels. Bandy is a winter team sport played on ice with similarities to soccer. Previous studies have investigated the physical workload during elite bandy by measuring heart rate and velocity patterns during match-play [1,2]. Blomqvist et al. [1] observed that elite bandy players reach a heart rate between 70–90% of their maximum and spend up to 27% of the time above the lactate threshold during match-play depending on the player’s position. Furthermore, Persson et al. [2] observed significant differences in the velocity patterns between defensive and offensive players, where defensive players spend significantly longer time skating between 4–20 km/h and the offensive players above 20 km/h.

Previous studies investigating the physical workload in bandy illustrate that bandy includes skating in velocities covering both aerobic and anaerobic intensities [1,2]. Activities at an anaerobic intensity, for example, acceleration, deceleration, and skating in high velocities, also contribute to the physical workload [3]. Global positioning system (GPS) devices are commonly used to quantify the occurrence and characteristics of accelerations, decelerations, and high-intensity movements during training and match-play in other sports [4]. To our knowledge, no study has investigated acceleration, deceleration and high-intensity skating during elite bandy match-play. Such data would provide further insight into the physical workload of the sport.

Acceleration and deceleration require high rates of force development that result in a higher metabolic cost and greater mechanical loading, soft tissue damage, and accumulated fatigue due to the high physiological load compared to constant velocity movements [4,5,6]. Excluding acceleration and deceleration in the estimation of physical workload will result in an underestimation since the profile would not account for the additional energy expenditure during transitions in velocity [7]. Quantification of acceleration and deceleration demands were previously carried out in other team sports such as soccer, rugby, field hockey, and ice hockey [5,8,9,10], but not in bandy. Bandy is similar to soccer in several ways—playtime, size of the plan, number of players on each team, and the rules. Both bandy and soccer are team sports with intermittent work and quick development of the play [1]. Previous studies on soccer have observed time-dependent reductions in both the total number and distance covered in acceleration and deceleration during competitive match-play [5,11]. These findings indicate that acceleration and deceleration capacity is acutely compromised during soccer match-play, which can result in both decreased performance and increased risk of injury [5].

Skating in bandy at velocities above 25 km/h is considered high-intensity skating [2]. A previous study observed differences between defensive and offensive players with offensive players (midfielders and forwards) spending more time at velocities above 25 km/h than defensive players [2]. However, knowledge is lacking regarding how high-intensity skating develops during bandy match-play. In football and rugby, it was observed that running at high velocities decreases over time by 20–25% from the first to the second half of the match [12,13,14]. Similar observations have been carried out in ice hockey, where the distance covered in a velocity above 24 km/h decreased from the first period to the last period of the match independent of player position [15]. According to Mohr et al. [16], the decline at the end of the match indicates a more permanent form of fatigue that can affect players’ performances. It is important to determine if similar patterns exist in bandy so that coaches and players are aware of profile changes in acceleration, deceleration, and high-intensity skating during match-play.

The aim of the current study was to elucidate further the physical workload in ten elite bandy players by describing acceleration, deceleration, and high-intensity skating during match-play. Based on the observations for similar sports in previous studies, we hypothesized that there would be a time-dependent reduction in acceleration, deceleration, and high-intensity skating.

## 2. Materials and Methods

### 2.1. Experimental Approach to the Problem

GPS (Catapult Innovations, Melbourne, Australia) was used to measure changes in total distance, two speed zones, acceleration, and deceleration during match-play. Data were collected from 10 elite bandy players during all 13 home matches in the 2016–2017 season. To determine performance changes during the match, a within-subject design was used to compare the first half of play to the second half. In addition, the match was divided into 15 min intervals to obtain a more detailed analysis of detecting fatigue in the various physical parameters. This design has been used previously for other sports [5,16,17].

### 2.2. Participants

Ten male elite bandy players participated in the study (age 30.0 ± 4.8 years; weight 82.8 ± 4.2 kg; height 180.6 ± 4.4 cm; body mass index 25.4 ± 1.3 kg/m^2^). The players were distributed as one libero, two defenders, two halves, three midfielders, and two forwards. The participants had at least five years of elite-level training and trained three to five times a week during the data collection period. In order for data to be included for comparison of the first and the second halves, players had to have played the whole match. Data were included in the 15 min interval analysis if players had played at least one half of the match. Because of illness (*n* = 2), injuries (*n* = 3) and technical problems (*n* = 5) some data were missing (*n* = 10) thus resulting in 120 observations (13 matches, 10 participants). The number of matches each player participated in was between 11 and 13 matches. Of those 13 matches, there were 11 wins, one draw, and one loss.

All players were informed orally and in writing about the study’s purpose and the procedures as well as their rights and risks of participating. Written consent was obtained from all participants prior to inclusion. The study was approved by the regional ethical review board in Uppsala, Sweden (Dnr: 2015/009), and was performed in accordance with the Declaration of Helsinki.

### 2.3. Procedures

Data collection was conducted between October 2016 and February 2017. Before beginning data collection, all participants were familiarized with the GPS unit as a part of their daily training activity. Before each match, there was a warm-up of about 20 min that consisted of aerobic exercises and movement training. The GPS unit was started by the players 15 min before the match. Participants wore the same GPS micro-technology unit (OptimEye X4, 50 × 90 mm, 67 g, Catapult Innovations, Melbourne, VIC, Australia) throughout the data collection. The GPS unit was placed in an elastic vest so that the unit was positioned between the shoulder blades of the thoracic spine. This was carried out to minimize interference on the unit [18]. The above procedures followed the manufacturer’s recommendation to obtain the most reliable measurements possible (Catapult Innovations, Melbourne, Australia). All home matches were played outdoors and the mean temperature was—3.7 °C ± 7.4 °C and wind speed was between 0 and 5 m/s.

A GPS receiver was set up and connected to the Catapult Sprint 5.1.2 software program (Catapult Innovations, Melbourne, Australia) to measure and monitor the recordings live. Substitutions, time-outs, and penalties were noted for use later in the analysis. The measurement started when the referee began each half and stopped when the referee ended each half. Data collection was continuous during both halves (2 × 45 min) and included substitutions, time-outs, and penalties. After the match, raw data files were downloaded and analyzed in Catapult Sprint 5.1.2 software. The data were later exported from Catapult Sprint 5.1.2 software into Statistical Package for the Social Sciences (SPSS, Windows Version 24.0, Inc., Chicago, IL, USA).

### 2.4. Global Positioning System Analysis

The micro unit captured GPS data with a sampling frequency of 10 Hz and 3D +/−16 g accelerometer data of 100 Hz. Johnston et al. [19] showed that the Catapult 10 Hz (OptimEye X4, 50 × 90 mm, 67 g, Catapult Innovations, Melbourne, VIC, Australia), GPS unit had a high intraclass correlation (ICC > 0.8).

The effect of the 90 min bandy match was investigated and divided into two halves (2 × 44:59 min:seconds) as well as in six 14:59 min:seconds intervals (I1 00:00–14:59, I2 15:00–29:59, I3 30:00–44:59, I4 45:00–59:59, I5 60:00–74:59, I6 75:00–89:59). Since substitutions and penalties occur in bandy, it was noted how many seconds each player was either substituted or was penalized for every 14:59 min:seconds interval. This variable was called off ice time (Table 1). Data collected during the extra time of the match were not included in the analyses.

The speed zones of 25–29.99 km·h^−1^ and ≥ 30 km·h^−1^ were based on previously defined published data from bandy [2] and recommendations by Sweeting et al. [20]. Acceleration and deceleration were defined as follows: (1) the acceleration/deceleration had to reach a minimum of 1 m·S^−2^ to mark the start of the acceleration/deceleration event; (2) the acceleration/deceleration had to reach and exceed the acceleration/deceleration limit of 2 m·S^−2^ to be counted as a high-intensity acceleration/deceleration; (3) the acceleration/deceleration had to reach and exceed the acceleration/deceleration limit of ≤ 3 m·S^−2^ to be counted as a very high-intensity acceleration/deceleration; (4) the acceleration/deceleration had to remain above this limit for at least 0.5 s; and (5) the acceleration/deceleration event ended when the acceleration/deceleration went below the minimum acceleration/deceleration speed of 1 m·S^−2^ (Table 1). The SprintTM software filtered the raw GPS velocity data using a median filter which is the default setting of the software (GPS Velsprint) (Catapult Innovations, Melbourne, Australia).

### 2.5. Statistical Analyses

The distribution of normality data was verified by using Shapiro–Wilk’s test, inspection of the skewness and kurtosis, and a visual inspection of the histograms, Q-Q plots, and box plots [21,22]. Data were expressed as the means ± standard deviation (SD). The paired samples *t*-test was used to determine the significance of differences between halves. A repeated-measures ANOVA was used to determine time effects between intervals, and a post hoc test Bonferroni was used to determine the significance of differences between I1 against the other intervals (I2, I3, I4, I5, I6). Cohen’s d effect size (ES) was calculated to establish the magnitude of difference between time intervals. The ES was categorized as small (0.2), medium (0.5) and large (0.8) [23]. The study used a significance level at *p* ≤ 0.05. All data were analyzed in the statistical program Statistical Package for the Social Sciences (SPSS) (Windows version 24.0, Inc., Chicago, IL, USA).

## 3. Results

### 3.1. First and Second Halves of Bandy Match-Play

The analysis showed that there was a significant decrease between the first and second half for total distance covered with 4.5%, very fast skating with 13%, sprint skating with 9%, and low-intensity acceleration with 3.5%. The other variables remained unchanged (Table 2). There was no significant difference for off ice time between the first and second half, but the large standard deviation was due to the exchanges being unevenly distributed between the players.

### 3.2. High-Intensity Work in 15 Min Intervals

For the total distance covered, there was a significant time effect, and I1 differed from I2, I5, and I6. For very fast skating, there was a significant time effect, and I1 differed significantly from all other intervals. For sprint skating, there was a significant time effect, and I1 differed significantly from I5. For low-intensity acceleration, there was a significant time effect, and I1 differed significantly from I2, I5, and I6. For low-intensity deceleration, there was a significant time effect, and I1 differed significantly between I2 and I5. No significant difference was observed between I1 and the other intervals for the other variables (Table 3).

## 4. Discussion

This study is the first to investigate differences in acceleration, deceleration, and high-intensity skating throughout match-play in bandy. The main findings are that no time-dependent reductions in high-intensity and very high-intensity acceleration or deceleration were observed during match-play, which is in contrast to the hypothesis. However, a significant time-dependent reduction was observed in the total distance covered in very fast skating, sprint skating, low-intensity acceleration, low-intensity deceleration, and total distance. This indicates that elite bandy players can maintain acceleration and deceleration at high intensities over time but skate a shorter distance both overall and at velocities above 25 km/h during the later stages of match-play.

In the current study, a significant time-dependent reduction was only observed for low-intensity acceleration, with a reduction of 3.5% from the first to the second half of the match. In soccer, several previous studies have observed a time-dependent reduction of 7.0–21.0% in accelerations for both high- and low intensities during match-play [5,11,24]. Time-dependent reductions in high-intensity activities, including both acceleration and deceleration, have also been observed in ice hockey between the first and third period of match-play [10]. Due to the similarities between sports, physical workload, and study design, the results regarding acceleration and deceleration will mainly be compared to soccer and not ice hockey [1,2]. The primary similarity between bandy and ice hockey is that both sports are performed on skates. The physical workload in ice hockey is characterized by shorter periods of work (20–90 s) with approximately half of the time spent at high-intensity skating [25]. This is in contrast to observations in bandy reporting up to one-quarter of the time spent at high-intensity skating. Moreover, the effective game time in bandy ranged between 70–90 min, compared to 15–25 min in ice hockey [2,25].

The differences in results between our study and previous studies in soccer may be explained by differences in the athletes’ training statues, method for movement analysis (motion analysis vs. GPS), and definitions of acceleration thresholds [11]. However, our study design is quite similar to that of Akenhead et al. [5] who analyzed acceleration in soccer, which implies that differences in the nature of the sports may contribute significantly to differences in results for acceleration profiles. One major difference between bandy and soccer is that bandy players skate instead of run. Skating involves a gliding phase where the players can rest while maintaining a high velocity; this is in contrast to soccer where players need to invest a large amount of energy to maintain their current velocity [2]. This suggests that bandy players have short periods of rest even when they are active in the match and skate at different velocities; whereas soccer players only rest when they are standing still. Another factor that differs between the sports is that bandy has an unlimited number of substitutions and soccer has a maximum of five. This implies that there are better conditions for recovery during match-play in bandy compared with soccer [26]. The short periods of rest and the unlimited number of substitutions may attribute to the finding in our study that bandy players can maintain high-intensity accelerations throughout match-play.

Our results show that there is no time-dependent reduction of high-intensity deceleration in bandy, but there is for low-intensity deceleration. Previous studies in soccer have observed time-dependent reductions in deceleration for both high- and low-intensity. This contributes to decreased performance and increased risk of injury since the players experience difficulties during rapid changes of directions [5,24]. A time-dependent reduction in deceleration has been explained by reduced central neural drive and accumulated eccentric load resulting in peripheral fatigue [5,27,28]. A recent study by Newans et al. [24] investigated the ratios of decelerations to accelerations in soccer and observed that the ability to decelerate in the moderate domain was maintained despite a decay in the ability to accelerate. These observations suggest that the time-dependent reduction in decelerations may not consistently be related to accumulated fatigue, but instead a lack of opportunity since a deceleration must be proceeded by an acceleration [24]. In the current study, there was a time-dependent reduction in both low-intensity deceleration and low-intensity acceleration when the match was divided into 15 min intervals. These results are in line with the findings of Newans et al. [24] since a reduction in deceleration was only observed together with a reduction in acceleration at the same intensity. As with acceleration, differences in the nature of sports may also affect the deceleration profiles, which may partly explain the differences between our results and findings in studies of soccer. One such difference between the sports is the movement patterns while changing direction. The quickest way of changing direction while running is to perform a high-intensity deceleration followed by an acceleration in another direction [29]. While skating, it is preferable to avoid a high-intensity deceleration and instead take a wider turn and use the turn to accelerate and build up a high velocity. Due to smaller decelerations and the use of the gliding phase while skating, it is possible that bandy players can change direction in a more energy-efficient way and therefore maintain both high-intensity acceleration and deceleration throughout match-play.

Findings in previous studies investigating physical workload in bandy indicate that skating at a velocity above 25 km/h is related to physical work on an anaerobic intensity for elite bandy players. This is based on the findings of Blomqvist et al. [1] and Persson et al. [2] who observed that offensive players (midfielders and forwards) spent more time above the lactate threshold than defensive players, and libero spent the least amount of time above the lactate threshold during match-play. Furthermore, forwards and midfielders spent more time ≥25 km/h and libero spent less time ≥25 km/h compared with the other player positions. The current study observed a significantly decreased distance covered at velocities ≥25 km/h, which is in line with previous studies in ice hockey [15,25]. This implies that the elite bandy players were unable to maintain the anaerobic work throughout an entire match since they covered a smaller distance on an anaerobic intensity. However, high-intensity acceleration is also considered as work on an anaerobic intensity [4], and no time-dependent reduction was observed in high-intensity and very high-intensity acceleration in the current study. There may be several explanations for a decreased amount of work on an anaerobic intensity in bandy. One possible reason is that the players experienced fatigue. Another possible reason is that the match development influenced the tactics, where disadvantage or advantage in the number of goals influences the need for higher or lower skating velocity [15]. However, Douglas et al. [10] point out the importance of high-intensity activities at the end of match-play for a greater probability of winning. Therefore, it can still be of importance to increase the players’ capacity for high-intensity skating and work at an anaerobic intensity to increase the likelihood of long-term success for the team.

When interpreting the current findings, a number of limitations should be considered. One limitation is that only one team was investigated. Therefore, it is possible that the outcome could be influenced by the teams’ tactics and individuals’ movement patterns. Future studies should investigate acceleration, deceleration, and high-intensity skating in different teams to investigate if the observations in the current study can be generalized for bandy at the elite level. The measurements in our study were collected with a 10 Hz GPS unit that can be considered a valid and reliable method for measurements in team sports and provides more accurate data than a 5 Hz GPS unit in measuring instantaneous velocity, distance covered in higher velocity, and elements of intermittent movement patterns [6,30,31]. It has been demonstrated that 10 Hz GPS units have some limitations regarding movements that are performed at velocities of 20 km/h or faster [19]. Another limitation with GPS is that the measurement cannot differentiate between forward and backward skating. However, it has been established that the 10 Hz system we used provides sufficient accuracy to quantify the acceleration and deceleration phases in team sports [5,6,8,11].

## 5. Conclusions

This study found no time-dependent reduction in high-intensity or very-high intensity acceleration and deceleration during elite bandy match-play. However, a time-dependent reduction was observed in the total distance covered of low-intensity acceleration and deceleration, high-intensity skating, and total distance. This may be explained by either fatigue or a change in tactics throughout the match-play. Bandy coaches should focus on increasing the players’ capacity for high-intensity skating to increase the ability for high-intensity activity throughout an entire match.

## Figures and Tables

**Table 1:** Operational definitions used to define global positioning system variables.

Variables	Operational Definition
Total distance covered	Distance covered (m) by all means of locomotion
Very fast skating distance covered	Total distance covered (m) in velocity between 25–29.99 km·h^−1^
Sprint skating distance covered	Total distance covered (m) at velocity ≥ 30 km·h^−1^
Low-intensity acceleration	* Distance covered (m) in acceleration between 1–1.99 m·s^−2^
High-intensity acceleration	* Distance covered (m) in acceleration between 2–2.99 m·s^−2^
Very high-intensity acceleration	* Distance covered (m) in acceleration ≥ 3 m·s^−2^
Low-intensity deceleration	* Distance covered (m) in deceleration between 1–1.99 m·s^−2^
High-intensity deceleration	* Distance covered (m) in deceleration between 2–2.99 m·s^−2^
Very high-intensity deceleration	* Distance covered (m) in deceleration ≥3 m·s^−2^
Off ice time	The time (s) the player was either substituted or penalized

* Minimal effort duration is defined as an individual acceleration/deceleration as an increase/decrease in speed for at least 0.5 s.

**Table 2:** Physical performance variables mean (with SD in parentheses) between the first and second halves of bandy match-play.

Variable	First Half	Second Half	*p*-Values	Effect Size (CI)
Total distance covered (m)	10,916.9 (1611.1)	10,450.3 (1738.2)	**<0.001**	0.278 (0.024–0.532)
Very fast skating distance covered (m)	1337.6 (681.5)	1160.9 (610.4)	**<0.001**	0.274 (0.020–0.528)
Sprint skating distance covered (m)	300.0 (205.3)	272.0 (201.5)	**0.017**	0.138 (−0.115–0.391)
Low-intensity acceleration (m)	342.7 (52.0)	333.0 (58.3)	**0.021**	0.163 (−0.09–0.417)
High-intensity acceleration (m)	83.8 (18.2)	83.2 (17.5)	0.639	0.034 (−0219–0287)
Very high-intensity acceleration (m)	48.2 (14.2)	49.7 (14.6)	0.171	0.104 (−0357–0.149)
Low-intensity deceleration (m)	219.7 (44.8)	218.6 (45.4)	0.742	0.024 (−0.229–0.277)
High-intensity deceleration (m)	49.1 (12.4)	49.8 (12.2)	0.488	0.057 (−0.196–0.310)
Very high-intensity deceleration (m)	50.5 (13.8)	50.7 (13.6)	0.863	0.015 (−0.238–0.268)
Off ice time (s)	338.2 (352.8)	341.8 (415.8)	0.879	0.009 (−0.262–0.244)

Significant difference (*p* ≤ 0.05) is marked in bold.

**Table 3:** Mean (with SD in parentheses) physical performance variables as a function of time throughout bandy match-play.

	Interval *						Time Effect	Effect Size	Difference Compared with I1
Variable	I1	I2	I3	I4	I5	I6	(*p*)	Cohen’s d	(*p* ≤ 0.05)
Total distance covered (m)	3796.0 (630.0)	3531.0 (636.8)	3616.3 (656.6)	3703.3 (709.9)	3394.1 (711.4)	3348.6 (807.9)	**<0.001**	0.62 (0.36–0.88)	I1 vs. I2, I5, I6
Very fast skating distance covered (m)	513.3 (24.9)	426.4 (21.4)	417.8 (21.4)	444.9 (22.7)	366.2 (19.7)	364.8 (21.9)	**<0.001**	6.33 (5.71–6.95)	I1 vs. I2, I3, I4, I5, I6
Sprint skating distance covered (m)	107.9 (76.7)	98.5 (80.0)	98.4 (84.2)	101.3 (94.0)	82.8 (72.6)	88.4 (82.8)	**0.005**	0.34 (0.08–0.59)	I1 vs. I5
Low-intensity acceleration (m)	118.0 (22.8)	110.0 (19.8)	115.5 (21.9)	118.9 (25.2)	106.3 (23.3)	107.9 (29.5)	**<0.001**	0.53 (0.28–0.79)	I1 vs. I2, I5, I6
High-intensity acceleration (m)	28.4 (7.6)	27.0 (7.7)	28.6 (7.5)	29.4 (8.5)	26.4 (6.8)	27.4 (7.9)	**0.003**	0.39 (0.13–0.64)	N/A
Very high-intensity acceleration (m)	15.9 (5.5)	16.0 (5.6)	16.4 (5.9)	17.2 (6.5)	16.4 (5.2)	16.1 (5.9)	0.205	1.58 (1.30–1.88)	N/A
Low-intensity deceleration (m)	76.6 (17.3)	71.2 (15.8)	74.4 (16.6)	76.1 (19.6)	71.5 (18.1)	71.1 (20.2)	**0.002**	0.29 (0.04–0.55)	I1 vs. I2, I5
High-intensity deceleration (m)	16.3 (5.4)	16.2 (5.1)	16.8 (5.3)	17.2 (5.1)	16.0 (4.8)	16.5 (5.9)	0.169	0.24 (−0.01–0.50)	N/A
Very high-intensity deceleration (m)	16.1 (6.0)	16.3 (6.1)	18.1 (6.5)	17.8 (6.3)	15.6 (5.2)	17.3 (6.7)	**<0.001**	0.42 (0.17–0.68)	N/A
Off ice time (s)	103.6 (136)	120.2 (140.6)	114.4 (146.8)	106.9 (157.9)	113.6 (164.8)	120.9 (191.5)	0.838	0.10 (0.15–0.36)	N/A

* I1 00:00–14:59, I2 15:00–29:59, I3 30:00–44:59, I4 45:00–59:59, I5 60:00–74:59, and I6 75:00–89:59. Significant difference (*p* ≤ 0.05) is marked in bold.

## Data Availability

The data presented in this study are available on reasonable request from the corresponding author.
